# Di­chlorido­{8-[2-(di­methyl­amino)­ethyl­amino]­quinoline-κ^3^ *N*,*N*′,*N*′′}zinc

**DOI:** 10.1107/S1600536813029929

**Published:** 2013-12-04

**Authors:** Abdul-Razak H. Al-Sudani

**Affiliations:** a Department of Chemistry, College of Science for Women, Baghdad University, Baghdad, Iraq

## Abstract

In the title complex, [ZnCl_2_(C_13_H_17_N_3_)], the coordination sphere of the zinc cation is distorted square pyramidal. The three N atoms of the *N*,*N*′,*N*′′-tridentate 8-[2-(di­methyl­amino)­ethyl­amino]­quinoline ligand and one chloride ion constitute a considerably distorted square base. The apical site is occupied by another chloride ion. The distortion from the ideal square-pyramidal geometry is manifested by the N—Zn—N angle of 133.25 (11)°. Like most square-pyramidal metal complexes, the zinc cation is displaced towards the apical chloride ion. In the crystal, mol­ecules are linked by N—H⋯Cl inter­actions. This leads to the formation of chains of mol­ecules parallel to the *b*-axis direction.

## Related literature   

For the role of the zinc cation in biochemical reactions, see: Xu *et al.* (2010 ▶); Jena & Manivannan (2012 ▶). For the geometry of five-coordinate zinc complexes, see: Dai & Canary (2007 ▶). For a related structure, see: Al-Sudani & Kariuki (2013 ▶).
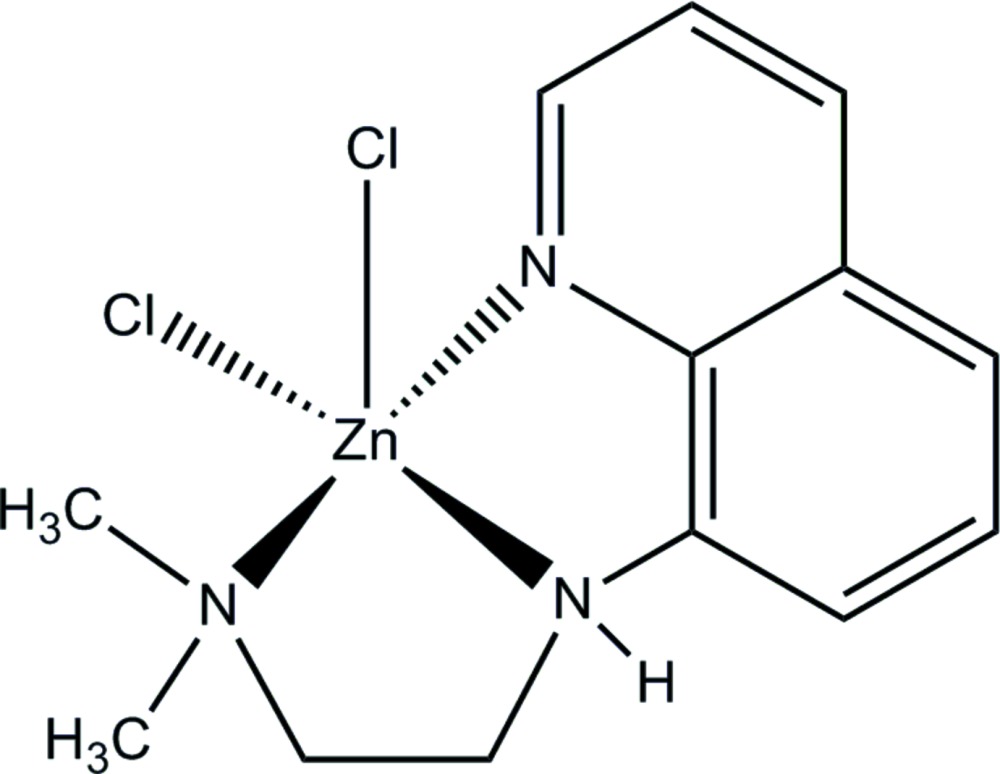


## Experimental   

### 

#### Crystal data  

[ZnCl_2_(C_13_H_17_N_3_)]
*M* *_r_* = 351.57
Orthorhombic, 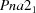

*a* = 23.6403 (6) Å
*b* = 7.5682 (2) Å
*c* = 8.0329 (3) Å
*V* = 1437.20 (8) Å^3^
*Z* = 4
Mo *K*α radiation
μ = 2.07 mm^−1^
*T* = 150 K
0.17 × 0.05 × 0.04 mm

#### Data collection  

Nonius KappaCCD diffractometer
Absorption correction: multi-scan (*DENZO*/*SCALEPACK*; Otwinowski & Minor, 1997 ▶) *T*_min_ = 0.720, *T*_max_ = 0.922
6701 measured reflections
2638 independent reflections
2422 reflections with *I* > 2σ(*I*)
*R*_int_ = 0.043

#### Refinement  

*R*[*F* ^2^ > 2σ(*F* ^2^)] = 0.030
*wR*(*F* ^2^) = 0.066
*S* = 1.07
2638 reflections
174 parameters
1 restraint
H-atom parameters constrained
Δρ_max_ = 0.31 e Å^−3^
Δρ_min_ = −0.38 e Å^−3^
Absolute structure: Flack (1983 ▶), 873 Friedel pairs
Absolute structure parameter: −0.002 (15)

### 

Data collection: *COLLECT* (Nonius, 2000 ▶); cell refinement: *SCALEPACK* (Otwinowski & Minor, 1997 ▶); data reduction: *DENZO* (Otwinowski & Minor, 1997 ▶) and *SCALEPACK*; program(s) used to solve structure: *SIR92* (Altomare *et al.*, 1993 ▶); program(s) used to refine structure: *SHELXL97* (Sheldrick, 2008 ▶); molecular graphics: *ORTEP-3 for Windows* (Farrugia, 2012 ▶); software used to prepare material for publication: *WinGX* publication routines (Farrugia, 2012 ▶) and *ACD/Chemsketch* (Advanced Chemistry Development, 2008 ▶).

## Supplementary Material

Crystal structure: contains datablock(s) I, New_Global_Publ_Block. DOI: 10.1107/S1600536813029929/hg5348sup1.cif

Structure factors: contains datablock(s) I. DOI: 10.1107/S1600536813029929/hg5348Isup2.hkl

Additional supporting information:  crystallographic information; 3D view; checkCIF report

## Figures and Tables

**Table 1:** Hydrogen-bond geometry (Å, °)

*D*—H⋯*A*	*D*—H	H⋯*A*	*D*⋯*A*	*D*—H⋯*A*
N2—H2*A*⋯Cl2^i^	0.93	2.65	3.420 (3)	141

Symmetry code: (i) 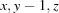.

**Fig. 1.**
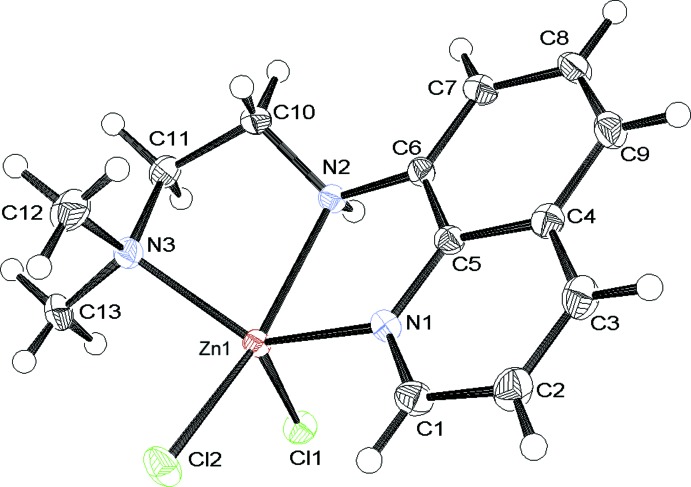
The asymmetric of (I) with atom labels and 50% probability displacement ellipsoids.

**Fig. 2.**
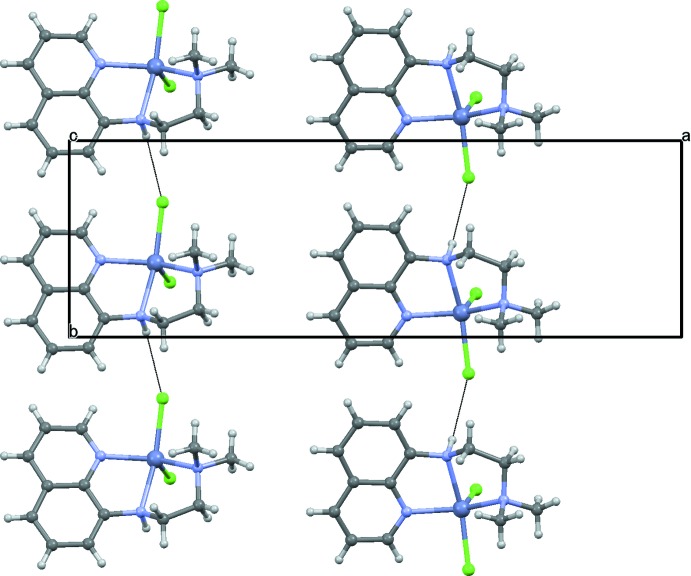
A segment of the crystal structure showing the N—H···Cl interactions as dotted lines.

## supplementary crystallographic information

### 1. Comment

Zinc is a biologically important element. Zinc, always present as a divalent cation in biological systems, is the second most abundant d-block metal ion in the human body after iron. The zinc cation; Zn^+2^, is well known to play diverse roles in many serious biochemical reactions, (Xu *et al.*, 2010; Jena & Manivannan, 2012). The zinc(II) ion, however, provides a number of coordination compounds because of its affinity towards different types of ligands and flexible coordination number ranging from two to eight. In zinc complexes, commonly found geometries are tetrahedral and octahedral. Six-coordinate complexes may be octahedral or trigonal-prismatic. Among the less common five-coordinate complexes, trigonal–bipyramidal geometry predominates over square-pyramidal geometry (Dai & Canary, 2007). Herein, we report the synthesis and characterization of 8-[2-dimethylamino]ethylamino]quinoline ZnCl_2_]. This complex was characterized by elemental analyses, mass spectroscopy, ^1^H-NMR, and single-crystal X-ray structure determination techniques. The single-crystal structure analysis of the complex reveals that the three nitrogen atoms of the tridentate ligand, *N*,*N*',*N*'', along with two chloride ions form a distorted square-pyramidal geometry around the zinc cation (Fig. 1). The three N atoms of the tridentate ligand, *N*,*N*',*N*'', and one Cl ion constitute a considerably distorted square base. The apical site is occupied by another Cl ion. The distortion from the ideal square-pyramidal geometry is manifested by the N1—Zn—N3 angle of 133.25 (11)°. As in most square-pyramidal metal complexes, the zinc cation is displaced towards the apical Cl ion. Zn—N2 bond [2.274 (3) Å] is longer than the other two Zn—N bonds [2.136 (3) and 2.138 (3) Å] and the Zn—Cl bonds are also differ in length [2.266 (1) for Cl1 and 2.360 (1) Å for Cl2]. The Cl2 ion and N2 atom are *trans* to each other with an N2—Zn—Cl2 angle of 158.87 (8)°. The molecules are linked by N—H···Cl interactions (Table 1). This leads to the formation of chains of molecules parallel to the *b* axis (Fig. 2).

### 2. Experimental

To a stirred methanoic solution (30 ml) of zinc dichloride (0.273 g m; 0.002 mol) kept under a positive nitrogen pressure, a methanoic solution (10 ml) containing an equimolar amount of the ligand (NN'N"); (0.43 g m; 0.002 mol) was slowly added. The resulting off white slurry was stirred at R·T. for 3 hrs. Then, the off white solid was collected by filtration, washed with smaller amount of cold methanol (5 ml, twice) followed by diethyl ether (15 ml, twice).the isolated solid was dried under vacuum at 50 °C. The mass of the slightly off white solid was 0.6 g m (yield = 85%), M·P. = 224°C. Mass Spec·(ES+)(CH_3_CN), m/*z* = 415.02 (*M*+CH_3_CN+Na) ^+^; 374.00 ((*M*+Na) ^+^; 314.04(M—Cl)^+^; 216.13(NN'N" + H)^+^. Anal·Calc. for [(C_13_H_17_N_3_) ZnCl_2_](M·W.:351.6); C; 44.41, H; 4.87, and N; 11.95. Found: C;44.45, H;4.9, and N;12.0. 1H NMR (d6-DMSO, 400 MHz, p.p.m.): 2.3 (S, 6H, N(CH_3_)_2_); 2.65 (t, 2H, CH_2_–CH_2_); 3.25 (t, 2H, CH_2_–CH_2_); 6.55 (broad s,1*H*, HN-quin.); the resonances of the other 6*H*-quin appear at 6.95, 7.3, 7.45, 7.6, 8.35,and 8.85.

Suitable colorless crystalof I were obtained *via* a slow vapor diffusion of diethyl ether in a smaller amount of acetonitrile solution of the zinc complex kept under the atmosphere of N2 gas.

### 3. Refinement

The N- and C-bound H atoms were geometrically placed (X—H = 0.95–0.99Å where X is N or C atom) and refined using a riding model with *U*_iso_(H) = 1.2–1.5*U*_eq_(X).

### Crystal data

**Table:**

[ZnCl_2_(C_13_H_17_N_3_)]	*F*(000) = 720
*M**_r_* = 351.57	*D*_x_ = 1.625 Mg m^−^^3^
Orthorhombic, *P**n**a*2_1_	Mo *K*α radiation, λ = 0.71073 Å
Hall symbol: P 2c -2n	Cell parameters from 2422 reflections
*a* = 23.6403 (6) Å	θ = 2.8–27.5°
*b* = 7.5682 (2) Å	µ = 2.07 mm^−^^1^
*c* = 8.0329 (3) Å	*T* = 150 K
*V* = 1437.20 (8) Å^3^	Block, colourless
*Z* = 4	0.17 × 0.05 × 0.04 mm

### Data collection

**Table:**

Nonius KappaCCD diffractometer	2638 independent reflections
Radiation source: fine-focus sealed tube	2422 reflections with *I* > 2σ(*I*)
Graphite monochromator	*R*_int_ = 0.043
CCD slices, ω and phi scans	θ_max_ = 27.5°, θ_min_ = 2.8°
Absorption correction: multi-scan (*DENZO*/*SCALEPACK*; Otwinowski & Minor, 1997)	*h* = −30→23
*T*_min_ = 0.720, *T*_max_ = 0.922	*k* = −9→9
6701 measured reflections	*l* = −8→10

### Refinement

**Table:**

Refinement on *F*^2^	Secondary atom site location: difference Fourier map
Least-squares matrix: full	Hydrogen site location: inferred from neighbouring sites
*R*[*F*^2^ > 2σ(*F*^2^)] = 0.030	H-atom parameters constrained
*wR*(*F*^2^) = 0.066	*w* = 1/[σ^2^(*F*_o_^2^) + (0.0224*P*)^2^ + 0.8402*P*] where *P* = (*F*_o_^2^ + 2*F*_c_^2^)/3
*S* = 1.07	(Δ/σ)_max_ < 0.001
2638 reflections	Δρ_max_ = 0.31 e Å^−^^3^
174 parameters	Δρ_min_ = −0.38 e Å^−^^3^
1 restraint	Absolute structure: Flack (1983), 873 Friedel pairs
Primary atom site location: structure-invariant direct methods	Absolute structure parameter: −0.002 (15)

### Special details

**Table:**

Geometry. All s.u.'s (except the s.u. in the dihedral angle between two l.s. planes) are estimated using the full covariance matrix. The cell s.u.'s are taken into account individually in the estimation of s.u.'s in distances, angles and torsion angles; correlations between s.u.'s in cell parameters are only used when they are defined by crystal symmetry. An approximate (isotropic) treatment of cell s.u.'s is used for estimating s.u.'s involving l.s. planes.
Refinement. Refinement of *F*^2^ against ALL reflections. The weighted *R*-factor *wR* and goodness of fit *S* are based on *F*^2^, conventional *R*-factors *R* are based on *F*, with *F* set to zero for negative *F*^2^. The threshold expression of *F*^2^ > 2σ(*F*^2^) is used only for calculating *R*-factors(gt) *etc*. and is not relevant to the choice of reflections for refinement. *R*-factors based on *F*^2^ are statistically about twice as large as those based on *F*, and *R*- factors based on ALL data will be even larger.

### Fractional atomic coordinates and isotropic or equivalent isotropic displacement parameters (Å^2^)

**Table:**

	*x*	*y*	*z*	*U*_iso_*/*U*_eq_	
C1	0.97979 (13)	1.0311 (4)	0.7265 (6)	0.0244 (7)	
H1	0.9611	1.1329	0.7685	0.029*	
C2	1.03902 (13)	1.0354 (5)	0.7106 (7)	0.0280 (8)	
H2	1.0595	1.1380	0.7422	0.034*	
C3	1.06704 (15)	0.8905 (5)	0.6491 (5)	0.0267 (9)	
H3	1.1069	0.8924	0.6350	0.032*	
C4	1.03549 (14)	0.7389 (5)	0.6071 (5)	0.0213 (8)	
C5	0.97605 (13)	0.7445 (5)	0.6265 (4)	0.0171 (7)	
C6	0.94322 (14)	0.5944 (5)	0.5872 (4)	0.0188 (7)	
C7	0.96889 (14)	0.4450 (5)	0.5276 (5)	0.0241 (8)	
H7	0.9467	0.3448	0.4992	0.029*	
C8	1.02845 (15)	0.4391 (5)	0.5079 (5)	0.0250 (8)	
H8	1.0458	0.3344	0.4673	0.030*	
C9	1.06105 (15)	0.5815 (5)	0.5466 (5)	0.0232 (8)	
H9	1.1009	0.5757	0.5332	0.028*	
C10	0.84685 (13)	0.5713 (5)	0.4719 (5)	0.0231 (8)	
H10A	0.8454	0.4428	0.4494	0.028*	
H10B	0.8617	0.6319	0.3718	0.028*	
C11	0.78850 (14)	0.6401 (4)	0.5145 (5)	0.0219 (8)	
H11A	0.7627	0.6197	0.4193	0.026*	
H11B	0.7735	0.5746	0.6116	0.026*	
C12	0.79589 (17)	0.9336 (6)	0.3988 (5)	0.0331 (9)	
H12A	0.8311	0.9008	0.3425	0.050*	
H12B	0.7966	1.0600	0.4252	0.050*	
H12C	0.7637	0.9083	0.3255	0.050*	
C13	0.73599 (14)	0.8808 (5)	0.6338 (5)	0.0283 (9)	
H13A	0.7348	1.0091	0.6497	0.043*	
H13B	0.7330	0.8219	0.7420	0.043*	
H13C	0.7044	0.8443	0.5627	0.043*	
Cl1	0.83558 (3)	0.77871 (12)	0.97688 (12)	0.02365 (19)	
Cl2	0.84770 (3)	1.18659 (10)	0.73045 (17)	0.02760 (19)	
N1	0.94882 (11)	0.8923 (3)	0.6858 (3)	0.0183 (7)	
N2	0.88348 (11)	0.6075 (4)	0.6168 (4)	0.0179 (6)	
H2A	0.8745	0.5245	0.6980	0.021*	
N3	0.79023 (12)	0.8311 (4)	0.5534 (4)	0.0199 (6)	
Zn1	0.859314 (13)	0.87704 (4)	0.71978 (6)	0.01701 (10)	

### Atomic displacement parameters (Å^2^)

**Table:**

	*U*^11^	*U*^22^	*U*^33^	*U*^12^	*U*^13^	*U*^23^
C1	0.0281 (15)	0.0196 (16)	0.0254 (17)	−0.0024 (12)	0.000 (2)	−0.002 (2)
C2	0.0242 (15)	0.0246 (18)	0.035 (2)	−0.0062 (13)	−0.002 (2)	−0.001 (2)
C3	0.0205 (16)	0.028 (2)	0.031 (2)	−0.0013 (15)	−0.0014 (16)	−0.0002 (18)
C4	0.0220 (16)	0.025 (2)	0.0166 (17)	−0.0018 (15)	0.0042 (14)	−0.0004 (16)
C5	0.0207 (15)	0.0140 (17)	0.0165 (16)	0.0013 (14)	0.0012 (14)	0.0005 (15)
C6	0.0187 (16)	0.0246 (19)	0.0131 (17)	0.0030 (15)	0.0007 (14)	0.0003 (14)
C7	0.0251 (18)	0.021 (2)	0.026 (2)	0.0027 (16)	0.0005 (16)	−0.0024 (16)
C8	0.0277 (18)	0.0224 (19)	0.025 (2)	0.0068 (16)	0.0032 (16)	−0.0026 (17)
C9	0.0220 (17)	0.026 (2)	0.0214 (19)	0.0028 (16)	0.0027 (15)	0.0012 (16)
C10	0.0215 (16)	0.0243 (19)	0.0236 (18)	0.0005 (14)	−0.0021 (17)	−0.0074 (18)
C11	0.0207 (17)	0.0186 (19)	0.026 (2)	0.0013 (14)	−0.0035 (15)	−0.0046 (16)
C12	0.040 (2)	0.034 (2)	0.025 (2)	0.0058 (19)	−0.0013 (19)	0.0083 (19)
C13	0.0190 (16)	0.032 (2)	0.034 (2)	0.0074 (16)	−0.0038 (16)	−0.0050 (19)
Cl1	0.0250 (4)	0.0253 (4)	0.0207 (4)	−0.0014 (4)	0.0019 (4)	0.0020 (4)
Cl2	0.0258 (3)	0.0148 (4)	0.0422 (5)	0.0004 (3)	0.0018 (6)	−0.0004 (6)
N1	0.0180 (12)	0.0160 (15)	0.021 (2)	−0.0015 (10)	−0.0003 (12)	0.0007 (12)
N2	0.0149 (13)	0.0185 (16)	0.0203 (15)	−0.0007 (12)	−0.0002 (12)	−0.0002 (12)
N3	0.0216 (14)	0.0205 (16)	0.0175 (15)	0.0039 (12)	0.0026 (12)	0.0011 (13)
Zn1	0.01713 (16)	0.01476 (18)	0.01915 (18)	0.00002 (13)	0.0011 (2)	−0.0008 (2)

### Geometric parameters (Å, º)

**Table:**

C1—N1	1.321 (4)	C10—H10A	0.9900
C1—C2	1.406 (4)	C10—H10B	0.9900
C1—H1	0.9500	C11—N3	1.480 (4)
C2—C3	1.373 (5)	C11—H11A	0.9900
C2—H2	0.9500	C11—H11B	0.9900
C3—C4	1.409 (5)	C12—N3	1.470 (5)
C3—H3	0.9500	C12—H12A	0.9800
C4—C5	1.414 (4)	C12—H12B	0.9800
C4—C9	1.421 (5)	C12—H12C	0.9800
C5—N1	1.376 (4)	C13—N3	1.484 (4)
C5—C6	1.412 (5)	C13—H13A	0.9800
C6—C7	1.369 (5)	C13—H13B	0.9800
C6—N2	1.436 (4)	C13—H13C	0.9800
C7—C8	1.418 (5)	Cl1—Zn1	2.2659 (11)
C7—H7	0.9500	Cl2—Zn1	2.3604 (8)
C8—C9	1.361 (5)	N1—Zn1	2.136 (3)
C8—H8	0.9500	N2—Zn1	2.274 (3)
C9—H9	0.9500	N2—H2A	0.9300
C10—N2	1.476 (5)	N3—Zn1	2.139 (3)
C10—C11	1.513 (4)		
			
N1—C1—C2	123.2 (3)	H11A—C11—H11B	108.0
N1—C1—H1	118.4	N3—C12—H12A	109.5
C2—C1—H1	118.4	N3—C12—H12B	109.5
C3—C2—C1	119.6 (3)	H12A—C12—H12B	109.5
C3—C2—H2	120.2	N3—C12—H12C	109.5
C1—C2—H2	120.2	H12A—C12—H12C	109.5
C2—C3—C4	118.7 (3)	H12B—C12—H12C	109.5
C2—C3—H3	120.6	N3—C13—H13A	109.5
C4—C3—H3	120.6	N3—C13—H13B	109.5
C3—C4—C5	118.4 (3)	H13A—C13—H13B	109.5
C3—C4—C9	122.6 (3)	N3—C13—H13C	109.5
C5—C4—C9	119.0 (3)	H13A—C13—H13C	109.5
N1—C5—C6	118.3 (3)	H13B—C13—H13C	109.5
N1—C5—C4	121.8 (3)	C1—N1—C5	118.2 (3)
C6—C5—C4	119.8 (3)	C1—N1—Zn1	124.1 (2)
C7—C6—C5	119.9 (3)	C5—N1—Zn1	117.6 (2)
C7—C6—N2	123.4 (3)	C6—N2—C10	115.7 (3)
C5—C6—N2	116.6 (3)	C6—N2—Zn1	111.7 (2)
C6—C7—C8	120.3 (3)	C10—N2—Zn1	107.8 (2)
C6—C7—H7	119.8	C6—N2—H2A	107.1
C8—C7—H7	119.8	C10—N2—H2A	107.1
C9—C8—C7	120.8 (3)	Zn1—N2—H2A	107.1
C9—C8—H8	119.6	C12—N3—C11	109.8 (3)
C7—C8—H8	119.6	C12—N3—C13	108.2 (3)
C8—C9—C4	120.1 (3)	C11—N3—C13	108.4 (3)
C8—C9—H9	120.0	C12—N3—Zn1	111.9 (2)
C4—C9—H9	120.0	C11—N3—Zn1	108.2 (2)
N2—C10—C11	107.0 (3)	C13—N3—Zn1	110.3 (2)
N2—C10—H10A	110.3	N1—Zn1—N3	133.25 (11)
C11—C10—H10A	110.3	N1—Zn1—Cl1	112.30 (8)
N2—C10—H10B	110.3	N3—Zn1—Cl1	109.10 (8)
C11—C10—H10B	110.3	N1—Zn1—N2	75.72 (10)
H10A—C10—H10B	108.6	N3—Zn1—N2	79.56 (10)
N3—C11—C10	111.0 (3)	Cl1—Zn1—N2	95.69 (8)
N3—C11—H11A	109.4	N1—Zn1—Cl2	93.79 (8)
C10—C11—H11A	109.4	N3—Zn1—Cl2	95.46 (8)
N3—C11—H11B	109.4	Cl1—Zn1—Cl2	105.31 (4)
C10—C11—H11B	109.4	N2—Zn1—Cl2	158.87 (8)

### Hydrogen-bond geometry (Å, º)

**Table:**

*D*—H···*A*	*D*—H	H···*A*	*D*···*A*	*D*—H···*A*
N2—H2*A*···Cl2^i^	0.93	2.65	3.420 (3)	141

Symmetry code: (i) *x*, *y*−1, *z*.
